# Mental Health Symptoms Associated with Sexualized Drug Use (Chemsex) among Men Who Have Sex with Men: A Systematic Review

**DOI:** 10.3390/ijerph182413299

**Published:** 2021-12-17

**Authors:** Daniel Íncera-Fernández, Manuel Gámez-Guadix, Santiago Moreno-Guillén

**Affiliations:** 1 Department of Personality, Assessment and Psychological Treatment, Autonomous University of Madrid, 28049 Madrid, Spain; manuel.gamez@uam.es; 2 Department of Medicine and Medical Specialties, Alcalá University, 28871 Alcala de Henares, Spain; smguillen@salud.madrid.org

**Keywords:** chemsex, slamsex, sexualized drug use, mental health, men who have sex with men

## Abstract

Background: Sexualized drug use (SDU), also known as chemsex, refers to the use of psychoactive substances for sexual purposes among men who have sex with men (MSM), which has been associated with mental health symptoms. The objective of this review is to systematically review the available evidence on mental health outcomes in MSM who use sexualized drugs. Methods: To prepare this systematic review, search strategies were developed and applied to the Web of Science, Science Direct, PubMed, and Scopus databases. A total of 117 articles were found, of which 12 were selected for the final review. Results: Those MSM who practiced SDU were more likely to experience from depression, anxiety, or a substance dependence, although these results were not found in all the studies analyzed. Among those who practiced the administration of intravenous drugs (referred to as slamsex), the mental health symptoms were more severe. Conclusions: This systematic review contributes to a fuller understanding of the mental health symptoms present in MSM who consume drugs for sexual purposes. Greater uniformity in data collection instruments is required, as well as the need to conduct a more in-depth assessment of the psychosocial adjustment of people who practice chemsex.

## 1. Introduction

In recent years, the use of psychoactive substances for sexual purposes among men who have sex with men (MSM), a phenomenon known as chemsex, and its implications on the physical and mental health has received increasing empirical attention [1,2,3]. Chemsex has been associated with goals such as enhancing sexual pleasure, facilitating sexual sessions that last several hours or days, and carrying out sexual fantasies and practices [4,5,6,7]. The chemsex phenomenon is also known as “party and play” (abbreviated as “PnP”) [8]; “intensive sex party” [9]; “session”, “sweets”, or “vice” [10]; “4/20” [11]; or “chill outs” [12]. These terms are used in different countries to define the idiosyncrasy of chemsex.

Previous studies have made it possible to draw up the sociodemographic profile of the chemsex participant. Users mostly identify themselves as gay or bisexual, their average age ranges between 32 and 42 years [1,2], most have completed university studies [13], have a high employment rate [14], and live in cities of more than 500,000 inhabitants [15].

Many MSM recognize that the popularity of chemsex and the easy availability of psychoactive substances is related to the use of geolocation social media applications such as Grindr, Scruff, or Hornet [12,16]. Moreover, MSM describe these internet platforms as important in this context [4,17], as their use can play a relevant role in facilitating drug use and risky sexual behavior [18]. While these encounters have been associated with private homes and hotel rooms, they are also commonly reported to happen in commercial sex establishments such as saunas or sex clubs [19,20,21]. The practice of chemsex in private domains can partly be attributed to the desire to adopt stigmatized and/or high-risk drug and sexual behaviors [12]. As such, private environments can be perceived as safe places and are accepted by men who engage in chemsex. The chemsex phenomenon is socially constructed, meaning it is subject to the preferences of those who practice it, their status, and the availability of specific drugs. These characteristics can vary over time and between cities and countries, and even between subcultures within countries [22,23,24]. Accordingly, Stuart [16] (pp. 9)defined chemsex as “syndemic of behaviors and circumstances”.

The most commonly used drugs for enhancing sexual experiences in MSM are the so-called “chemsex substances” [17,25]. These include gamma-hydroxybutyrate/gamma-butyrolactone (GHB/GBL or “liquid ecstasy”), 4-methylmetcathinone (mephedrone), N-methyl-1-phenylpropan-2-amine (methamphetamine or “crystal”), and ketamine [8,17,26,27,28]. However, other more traditional sexualized drug use (SDU) may be involved in chemsex practices. These drugs include cocaine, 3,4-methylenedioxy-methamphetamine (MDMA or “ecstasy”), alkyl and butyl nitrites (poppers) [24], erectile dysfunction medications, and cannabis [2]. A significant number of MSM participating in chemsex use three or more drugs before or during sex sessions [14,29], not always being chemsex substances the chosen ones [15,30]. For example, Torres et al. [30] found that cocaine was the most used substance before or during sexual intercourse in their study. In the same way, Stevens et al. [31] found that alcohol was the most widely used substance among chemsex-related drug users. While most MSM who engage in chemsex to improve their sexual relationships administer drugs non-intravenously, some administer drugs intravenously; this practice is known as slamsex or slamming [12,14]. Chemsex has been associated with both mental health and sexual health risks [8,27,32].

Evidence suggests that chemsex participants present a greater probability of engaging in risky sexual behavior [5,12,17,31,33,34,35,36], which has been associated to several negative outcomes. These outcomes include sexually transmitted infections including the human immunodeficiency virus (HIV) [13,37] and various mental health outcomes including compulsive sexual behavior, addiction, and mood disorders [38].

Although mental health of people who use drugs for sexual purposes have started to be investigated in recent years, mental health outcomes has been less studied to date [39]. There is available evidence showing that drug use for sexual purposes has been associated with mental health symptoms [27,40]. For example, Tomkins et al. [2] noted in their systematic review that chemsex is associated with depression, anxiety, and psychotic symptoms. In his recent study, Tan et al. [41] indicated that the participants described positive and desired aspects associated with SDU, but they also described how SDU was used as a coping mechanism to deal with emotional and situational triggers, such as HIV-related stigma, racism, sexual violence, death and loss, neglect, and internalized homophobia. However, there is a limited amount of data available about the severity of mental health among persons that participate in chemsex.

Mental health symptoms among people who practice chemsex may be due to several reasons, including prejudice, discrimination, and social stigma as a source of long-term stress [42,43,44,45]. According to Burton et al. [46] people belonging to sexual minorities experience from reduced psychological health compared to heterosexual individuals. This has been associated with formal factors (such as legal sanctions) or informal factors (such as victimization). Young people experiencing victimization report higher levels of depression, suicidal ideation, suicide attempts, substance use, and absenteeism [47,48]. Minority stress theory [44] provides a framework for understanding psychological outcomes among sexual minorities. This theory states that sexual minorities are chronically exposed to different stressors such as harassment and victimization, rejection expectations, escape and hiding, prejudices, and the risk of suffering violence simply because they are a sexual minority [49,50]. These pressures could affect the mental health and wellbeing of gay and bisexual men, and could be exacerbated by reductions in social support to help face them [51]. Potential mental health symptoms among people who use drugs for sexual purposes could be associated with increased vulnerability to sexually transmitted infections (STIs) and a possible reduction in the effectiveness of prevention efforts prior to HIV infections [52].

The objective of this systematic review was to expand the excellent previous reviews, analyzing a wider range of problems, including anxiety, depression, substance dependence, or general mental health among persons who uses drugs in a sexual context (including slamsex). This review will help to clarify the currently identified mental health problems associated with drug use for sexual purposes and will propose some recommendations to improve psychological assessment and intervention among people who practice chemsex.

## 2. Materials and Methods

### 2.1. Protocol and Research Question

This review was carried out according to the Preferred Reporting Items for Systematic Reviews (PRISMA) [53]. The chosen PRISMA-P version [54] consists of a 27-item checklist intended to facilitate accurate and reliable preparation and reporting for a systematic review. To ensure the quality of the study, this review followed the seven steps of Cooper’s model [55]: (1) Formulation of the review problem; (2) Scientific literature search; (3) Compilation of information from the studies consulted; (4) Evaluation of studies quality; (5) Analysis and integration of results of the selected studies; (6) Interpretation of the evidence; and (7) Presentation of results.

To determine the research question, the population, intervention, and outcome (PIO) components proposed by the Joanna Briggs Institute [56] for systematic reviews of etiology and risk studies were used. These components are P (men who have sex with men), E (chemsex), and O (mental health outcomes). Therefore, the question for the systematic review was as follows: What is the existing scientific evidence on mental health symptoms present in MSM who practice chemsex?

### 2.2. Eligibility Criteria

One of the inclusion criteria for the scientific articles was that the investigation should study the use of substances for sexual purposes among MSM. However, only those that described some relationship between mental health and SDU/chemsex were considered. This criterion allowed to discard papers on the use of recreational drugs in other contexts (such as music festivals, friends, and parties). Studies that analyzed the use of drugs administered intravenously before or during sexual relationships were also considered. Studies investigating participants with both HIV-positive and HIV-negative serological status were also included, as the review focused on MSM regardless of their serological status. Studies published in indexed journals between 2010 and 2020 were included. This review includes research among MSM participants over 16 years old and publications written in English, Spanish, or French, without any restriction of country of origin where the study was conducted.

By applying exclusion criteria, theoretical or conceptual papers (such as case reports, reviews, and book chapters), letters to the editor, conference proceedings, and qualitative researches were all excluded. Further, studies whose population was not MSM (such as lesbian women or swingers) were also excluded because they were not considered within the objectives of this review.

### 2.3. Information Sources and Search Strategy

A systematic search of scientific literature was carried out through electronic databases (Web of Science, Science Direct, PubMed, and Scopus), and the bibliographic references lists of the included articles were examined as a manual search strategy to prevent any loss of relevant information. A literature search plan was performed by combining the following key words and Medical Subject Heading (MeSH) terms: “men who have sex with men”, “gay man/men”, “bisexual man/men”, “homosexual man/men”, “sexualized drugs”, “sexualized substance”, “slamsex”, “party and play”, “chemsex”, “mephedrone”, “cathinones”, “n-methyl-3,4-methylenedioxyamphetamine”, “MDMA”, “GHB”, “gamma hydroxybutyrate”, “cocaine”, “poppers”, “gamma methamphetamine”, “Viagra”, “gamma hydroxybutyric acid”, “ketamine”, “psychological disorders”, “psychiatric disease”, “mental disorders”, “mental health”, “mental health symptoms”, “mental health outcomes”, “psychological wellbeing”, “anxiety”, “depression”, and “addictive behavior”. All terms were searched individually or in combination using the Boolean connectors AND and OR within the Title, Abstract, and Keywords categories. The database search was conducted between February and May 2020, and the implemented search strategy can be seen in Table 1.

### 2.4. Coding of Studies

The selected papers were analyzed in order to understand the relationship between chemsex and mental health in MSM. Chemsex was defined as the intentional use of drugs (cannabis, cocaine, poppers, medication for erectile dysfunction, MDMA, GHB/GBL, methamphetamine, mephedrone, ketamine) for sex. A broad definition of substances used for sexual purposes was used as, according to the existing literature, substances such as cannabis, poppers, and cocaine have also been associated with the practice of chemsex [57,58]. Subsequently, the full content of papers was analyzed to extract the information from each one, which was then coded in an Excel sheet for subsequent analysis and discussion. A list was made with the criteria chosen to assess the quality of studies. This included author(s), date of publication, study where the article is nested, year(s) of data collection, country in which the research was carried out, journal where the results were published, study objectives, type of study, sampling, sample origin, sample size, data collection instruments, and main results.

## 3. Results

### 3.1. Study Selection

**Figure 1:**
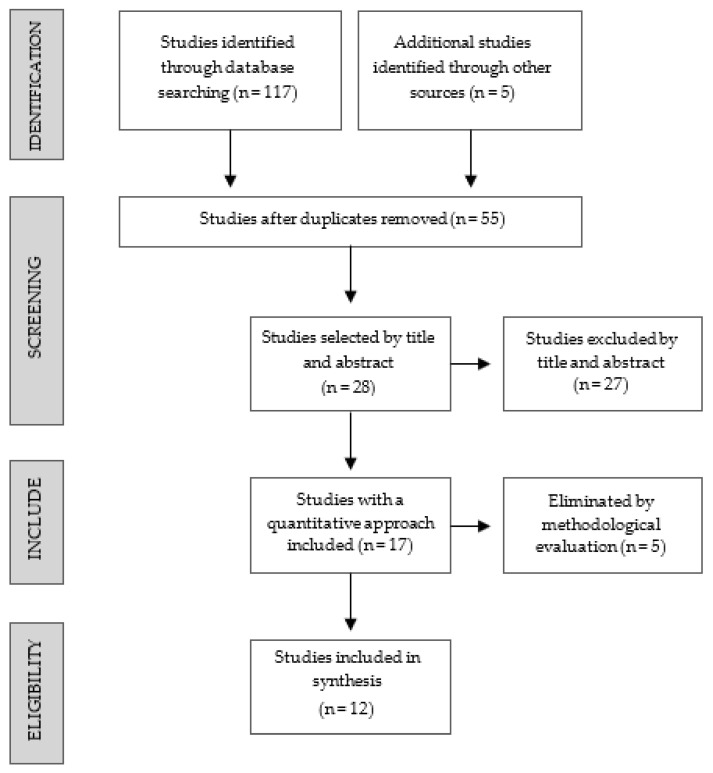
Study selection process according to the PRISMA Diagram.

The articles used in this review were selected using the PRISMA Search Diagram [54], and the study selection process can be seen in Figure 1. A total of 117 potential studies were initially found after applying the search strategy. Bibliographic reference lists of the selected studies were screened to locate additional studies, resulting in five more articles that were incorporated in the search. Duplicated articles were then eliminated (n = 67), and 28 of the remaining 55 articles were selected based on title and abstract. Seventeen studies with a quantitative approach were included. The five studies that did not explicitly analyze participants’ mental health were discarded. This resulted in 12 studies meeting the requirements for inclusion in a systematic literature review. Descriptive characteristics of the included studies can be seen in Table 2.

### 3.2. Prevalence and Type of Drug Use in Chemsex

Regarding chemsex practice, drug use before or during sexual encounters was analyzed in all selected studies. In 10 of the papers (83.33%), the term “chemsex” was explicitly mentioned on some occasions [14,22,58,59,62,63,64,65,66,67]. The remaining publications used the expressions “use of sexual drugs” [60] and “use of recreational substances commonly used during sexual activity” [61] when referring to the same phenomenon. Estimates of variation in chemsex prevalence ranged between 5.8% and 90% considering all the papers included in the review. The highest and lowest prevalence were found in the studies by Batisse et al. [59] and Brogan et al. [22], respectively. Regarding the period of time studied, chemsex practice was analyzed by two studies (16.67%) in the previous 3 months [14,65], four (33.33%) in the previous 6 months [22,58,60,63], and four (33.33%) in the previous 12 months [62,64,66,67], while two (16.67%) studies did not provide information about the time period [59,61]. Some of the analyzed studies described the different substances used by participants, including cannabis, poppers, GHB/GLB, mephedrone, methamphetamine, ketamine, MDMA, erectile dysfunction drugs, and cocaine. Five of the studies (41.67%) also examined recorded prevalence of these drugs when they were administered intravenously [22,59,62,67]. The drugs used by participants in the different studies were recruited from different groups: two studies (16.67%) only recorded drugs consumed by those who practiced slamsex [59,67], one study (8.33%) recorded the substances consumed by those practicing chemsex [62], two studies (16.67%) referred to substances that methamphetamine users ingested [66] or poppers [58], and five studies (41.67%) examined drugs consumed by the entire sample, regardless of whether they practiced chemsex [14,22,60,61,63]. The prevalence percentages of each substance are listed in Table 3.

### 3.3. Assessment Techniques

With regard to the data collection systems, most of the studies (58.33%) used questionnaires or self-administered online interviews [22,58,61,62,63,64,66]. In addition, one study (8.33%) used a self-administered paper-and-pencil questionnaire [14], two (16.67%) used self-administered questionnaires on tablet [65,67], one (8.33%) used a combination of self-administered questionnaire and interview [60], and one (8.33%) used clinical observations recorded in various pharmacoepidemiological tools [59]. A total of 30 scales or subscales were used to evaluate mental health outcomes of MSM. Further, questions about previous psychiatric diagnoses, self-reported current psychological symptoms, and self-reported psychological antecedents were recorded. The psychological assessment instruments included in the studies are detailed in Table 4.

It is remarkable that most of instruments used in this study only measure the mental health of participants generally (i.e., Rusell at al. [68] and Lin et al. [69]). The most commonly used questionnaire in the analyzed studies was the Patient Health Questionnaire (PHQ) [70], which was used in six investigations [14,22,58,63,65,66]. The PHQ is one of the most widely used tests in the context of international primary care [71]. Moreover, it is a screening tool derived from the self-report test of the PRIME-MD system, an instrument for the evaluation of mental health used in primary care. The PHQ contains several modules that evaluate symptoms anxiety (GAD-7), symptoms depression (PHQ-9), somatic symptoms (PHQ-15), and symptoms panic (PHQ-PD). In addition, a short test derived from the PHQ (PHQ-4) is included for anxiety and depression. The PHQ-9 allows identification of mild, moderate, or even severe depressive symptoms. The PHQ-9 consists of nine items that assess the presence of depressive symptoms (corresponding to the Diagnostic and Statistical Manual of Mental Disorders criteria) present in the previous 2 weeks [71].

The Alcohol Use Disorders Identification Test (AUDIT) is a self-administered screening questionnaire created by Saunders et al. [72]. It was used in two of the investigations in this review [14,61] to identify dangerous and harmful patterns of alcohol consumption among participants during the previous year [73,74]. AUDIT consists of 10 questions: the first three refer to risky use, the next three examine possible symptoms of dependence, and the final four assess harmful alcohol use [72]. AUDIT-C was the version chosen in the two studies. It is an abbreviated version that groups the first three questions as follows: “How often do you consume any alcoholic beverages?” “How many times do you usually consume alcoholic beverages in a normal drinking day?” and “How often do you drink six or more alcoholic beverages in a single day?” The sensitivity and specificity confirm the validity of this questionnaire for identifying risky alcohol consumption [75].

The Kessler Psychological Distress Scale (K-10) was created by Kessler et al. [76]. It is an easily applied screening assessment instrument that was used to assess risk of experiencing from nonspecific psychological distress in two of the reviewed studies [61,64]. Further, K-10 comprises 10 questions on emotional states that assess the presence of symptoms of depression and anxiety in the previous month.

In addition to the questionnaires, self-reported measures were recorded as questions about previous psychiatric diagnoses and current psychological symptoms [39], in addition to psychological history [59]. Demant & Oviedo-Trespalacios [61] applied ten psychological assessment tests and questionnaires, being the study that used the most instruments, while three investigations only used a psychological evaluation questionnaire including self-reported measures of psychological history [59,65,67].

### 3.4. Mental Health

According to the data obtained, nine of the studies analyzed (75%) indicated there was a relationship between chemsex and mental health outcomes [14,22,59,60,62,64,65,66,67]. Depression was the most frequent outcome among the studies, with six (50%) finding a positive association between chemsex and depression symptoms [14,22,62,65,66,67]. Further, four studies (33.33%) found a positive relationship between chemsex and anxiety symptoms among the participants [22,59,60,62]. Meanwhile, three articles (25%) established a positive association between practicing chemsex and suicidal ideation [22,59,62]. Two of the publications (16.67%) also related chemsex with being dependent on some substance and presenting psychotic symptoms [59,62].

### 3.5. Relationship between Non-Intravenous Drug Use and Mental Health

Among the studies that analyzed non-intravenous chemsex, Brogan et al. [22] found that 24% of participants exhibited moderate to severe levels of anxiety and depression, and 26% revealed occasional suicidal thoughts (almost every day in the previous 2 weeks). In their longitudinal study, Nöstlinger et al. [65] identified that 12% of participants exhibited moderate to severe levels of depression at baseline, which increased to 15% and 16% at 9 and 18 months, respectively. In addition, six serious adverse events related to mental health outcomes and/or drug use were documented throughout the study period: one study participant died due to a GHB overdose, one committed suicide after an alcohol and drug overdose, and four participants reported being diagnosed with some psychiatric disorder. Another study found that methamphetamine users exhibited significantly higher levels of depression compared to men who did not use drugs before or during sex [28]. Sewell et al. [14] found that depressive symptoms were present in 33% of non-chemsex drug users and in 29.2% of chemsex drug users. This research also associated an increased risk of drinking alcohol among those who used chemsex drugs or recreational drugs compared to those who did not use any drugs. Depressive symptoms and an increased risk of alcohol use were associated with chemsex drug use and the use of three or more recreational drugs in the previous 3 months. Finally, Hibbert et al. [64] noted that MSM who participated in chemsex were more likely to report lower levels of life satisfaction than those who did not practice chemsex. However, no significant differences were identified on this issue between those who used chemsex substances and those who used other types of SDU.

### 3.6. Relationship between Intravenous Drug Use and Mental Health

Regarding the mental health outcomes associated with the practice of slamsex, Dolengevich-Segal et al. [62] noted that people who had participated in slamsex were more likely to report current self-reported psychiatric disorders than those who participated in non-intravenous chemsex. Compared to participants who engaged in non-intravenous chemsex, those who engaged in slamsex were more likely to report depressive symptoms (61.8% versus 28%), anxiety symptoms (47.1% versus 23.1%), and addictive symptoms (38.2% versus 15.4%). Similarly, Batisse et al. [59] revealed an association between slamsex and psychiatric disorders (i.e., psychotic symptoms, agitation, anxiety, or suicide attempts) in 50% of cases, acute intoxication in 25% of cases (which included three deaths), dependence and abuse in 17% of cases, and seroconversion in 8% of cases. Trouiller et al. [67] found that slamsex participants reported poorer mental health and higher antidepressant use over the previous 12 months than those who did not participate in slamsex. The mental health score (mean, 0 to 100; 100 = best possible state) among those who had never slammed was 66.5 versus 51.8 who slammed. Furthermore, a strong association was observed between slamsex and seropositivity to both HIV and hepatitis C virus (HCV). HIV seroprevalence was four times higher (48.8% versus 13.4%) among people who reported participating in slamsex.

### 3.7. Absence of Relationship between Drugs and Mental Health

Three of the studies examined (25%) found no relationship between chemsex practice and mental health outcomes [58,68,69]. Although popper consumption was high, as highlighted in the research by Demant & Oviedo-Trespalacios [61], incidences of dependence symptoms and risky consumption were not significant. However, association was found between recent popper use and the use of other psychoactive substances. Hammoud et al. [63] found no statistically significant associations between GHB use (ever or recently) and elevated scores on depression and anxiety measures. In fact, MSM who used GHB in the previous six months were less likely to report symptoms of depression than those who did not engage in SDU. However, men who had used GHB at least once a month or more in the previous 6 months (compared to those who had used it less than once a month) reported greater physical health outcomes as a result of the drug use. These outcomes included needing medical attention, having an accident, and major social complications. In another study, Vaccher et al. [58] also discovered that men who had used poppers (both in the past and more recently) scored lower on measures of depression and anxiety than men who had never used them. However, men who had used poppers frequently reported a greater number of sexual partners. Further, they were more likely to have engaged in group sex (30.4% versus 12.4%), practiced chemsex (26.3 % versus 2.5%), and engaged in sex with casual partners (42.1% versus 24.1%) over the previous 6 months compared to men who had never used poppers.

## 4. Discussion

The purpose of this review was to analyze and synthesize the information available on chemsex and its relationship with mental health in MSM. Twelve studies with a quantitative approach were selected and reviewed to analyze results that associated chemsex practice with psychological adjustment. Despite the limited mental health information pertaining to people who use drugs for sexual purposes, the findings in this review indicate that chemsex is associated in nine out of twelve studies with worse mental health, including depression, anxiety, and dependence on drugs or other substances.

Findings in studies regarding drug use during sex indicate that MSM more frequently use so-called “chemsex substances” (GHB/GBL, mephedrone, methamphetamine, and ketamine). However, participants also described the use of more traditional recreational drugs involved in this practice, such as cocaine, MDMA, poppers, erectile dysfunction medications, and cannabis. This review highlights that poppers are the most widely used drugs in half of the analyzed studies. This may be due to the relatively easy accessibility of poppers [61]. Heterogeneity in the use of drugs for sexual purposes in the reviewed studies can be partly explained by the fact that the investigations analyzed the use of different types of drugs (such as chemsex substances, methamphetamine, and poppers) as well as by the study populations and sampling techniques.

The results of this review indicate that frequent use, the use of increasingly higher doses, the route of administration, and the combination of different drugs may be associated with mental health symptoms (such as anxiety and depression). Even the consumption of substances used in sexual contexts with a considerably lower risk profile (such as poppers) can be related to the use of other more dangerous psychoactive substances (chemsex drugs). Future studies should consider the influence of alcohol consumption in the genesis and maintenance of the consumption of chemsex substances. Alcohol could promote facilitating or disinhibiting effects on chemsex, which could lead to risky sexual behavior.

The frequent practice of slamsex was associated with more risky sexual behaviors and the use of multiple drugs. Further, slamsex participants were diagnosed with some STIs and mental health symptoms more frequently than those who did not inject drugs [59,62,67]. This may be due, in part, to needle and syringe exchanges between slamsex participants, as indicated in previous research [59,67]. Intravenous drug use among those engaging in slamsex could be associated to risky sexual behavior and high HIV seroprevalence. This establishes the need for specific intervention strategies based on the prevention of STIs in these contexts.

Many MSM may perceive drug use in the sexual sphere as common and normalized behavior [17,77,78]. However, the degree and frequency of psychoactive substance use before and/or during sex sessions can vary among MSM. There are significant differences in the prevalence of chemsex usage reported in different studies, ranging between 5.8% and 90% in our review. These data are consistent with the review by Tomkins et al. [2], where they identified a prevalence range of between 4% and 94%. A longitudinal analysis of drug use in MSM in Belgium reported that chemsex practice had been stable among participants (45% to 46%) over a period of 18 months. However, these data do not match other researches where there was an increase in the general use of chemsex drugs over a 10-year period [79]. Among those who practice slamsex, prevalence is associated with a minority of MSM who practice chemsex. However, slamsex participation ranged between 2% and 61% in our review. These findings do not allow us to conclude that slamsex has such a high prevalence. Rather, these data are due to one of the publications [59] focusing almost exclusively on studying intravenous drugs for sexual purposes in MSM.

The outcomes of this systematic review reflect a greater tendency for the study of depression, anxiety, and dependence on, or addiction to, drugs among MSM who consume any drug for sexual purposes. Other possible mental health symptoms associated with chemsex among MSM were less analyzed, such as psychiatric history, suicidal ideation, current self-reported psychiatric disorders, psychotic symptoms, somatic symptoms, post-traumatic stress, low self-esteem, or a negative impact on life. These findings coincide with the results of Tomkins et al. [2] and Maxwell et al. [1], where the use of drugs for sexual purposes was reported to be associated with physical and mental health symptoms. Studies identified a greater number of mental health symptoms associated with chemsex practice among slamsex practitioners, which has also been identified in literature reviews of psychiatric and addictive symptoms [79].

There is substantive evidence in this review that highlights the relationship between substance use in a sexual context and depressive symptoms. This association is most consistent among the different recorded mental health symptoms, regardless of whether they were longitudinal or cross-sectional studies (with an HIV positive or HIV negative population), whether they practiced slamsex, or whether the studies analyzed a single drug or focused on several substances. These results agree with other studies where users of different drugs had a significantly higher prevalence of depression symptoms [78,80]. This association between drug use and depression could be moderated by the type of substances ingested. For example, dependent methamphetamine users, those who used cannabis, those who smoked tobacco daily, and those who were classified as high or severe risk drinkers were more likely to present depression symptoms compared to non-users. In contrast, those who consumed poppers or GHB did not show symptoms of depression. In fact, those who used GHB had better scores on mental health tests than those who did not use the substance. These findings seem to indicate that mental health of those who use chemsex substances and those who participate in other types of SDU is similar. However, other factors (such as the route of administration, frequency of consumption, and amount ingested) should be considered to establish this association with more authority. For example, the study by Dolengevich-Segal et al. [62] indicated that the genesis or maintenance of mental health outcomes also depends on the route of administration of the drug. Further, MSM who were HIV positive and participated in slamsex exhibited higher rates of symptoms related to depression, anxiety, dependence drug, paranoid ideation, and suicidal ideation compared to those who ingested non-intravenous drugs for sexual purposes.

Symptoms of anxiety among people who practiced chemsex were reported by three of the studies reviewed. Card et al. [60] indicated that cognitive escape is associated with high levels of trait anxiety and the search for sexual sensations. This could be explained by people using drugs strategically as a way of meeting social expectations and of coping with other sources of anxiety. It could also be due, at least in part, to the fact that social support is negatively associated with the intention of using drugs, which could generate symptoms of anxiety due to the pressure to not consume such substances [81]. As in depression, some substances are more likely to generate different anxiety symptoms. Note that recent methamphetamine users (a significant percentage classified as dependent) exhibited increased evidence of anxiety compared to those not classified as dependent. This is consistent with other research, where dependence on drugs was associated with high anxiety levels [82,83]. On the other hand, intravenous drug use for sexual purposes among MSM was significantly associated with high anxiety symptoms; this association could be related to slamsex practice. A self-reported current anxiety symptom was more common among participants practicing slamsex than in those practicing non-intravenous chemsex.

In relation to studies that established an association between drug use before or during sexual practices and symptoms of depression and anxiety, note that these symptoms could be related to other mental health outcomes. For example, in the Canadian EMIS-2017 study developed by Brogan et al. [22], they found that the frequency of suicidal ideation and possible alcohol dependence was associated with the reported high levels of anxiety and depression. This could partly be because the association between chemsex and depression or anxiety could be influenced by dependent drug use. However, our findings show that most of men who used drugs were not necessarily dependent users, nor was their use always considered problematic. This review indicates that high levels of dependence or addiction to drugs are mainly associated with those who use intravenous drugs for sexual purposes as the route of administration. This allows us to infer that the route of administration is associated with the symptoms of abstinence and dependence symptoms. Furthermore, referring to this same issue, we must mention the study by Dolengevich-Segal et al. [79], which was not included in the present review. They indicated higher levels of abstinence and dependence among MSM who injected drugs for sexual purposes compared to those who participated in non-intravenous chemsex.

It is well known that depression is related to a greater tendency to take more sexual risks [84,85]. Our findings agree that participation in chemsex is associated with risky sexual behavior. This finding is coincident with other studies that found anxiety and depression symptoms to be associated with risky sexual behavior in the heterosexual [84] and homosexual [86] populations.

This review indicates that chemsex may be associated with other disturbances, such as suicidal ideation, psychotic symptoms, current or past self-reported psychiatric disorders, or a negative impact on life. However, it is difficult to reach a definitive conclusion with such limited evidence. The findings found in these studies do not allow us to conclude that practicing chemsex in itself is a risk to poorer mental health. This may be partly due to the question of whether these mental health outcomes are directly related to chemsex practice or, to some extent, represent prior vulnerabilities. It would be important to determine if the mental health outcomes are present before exposure to chemsex and what events are related to its genesis and maintenance. For example, it is known that sexual minorities (people with a homosexual or bisexual sexual orientation) experience worse mental health outcomes compared to the heterosexual population. Further, they present higher rates of mental health symptoms, as revealed by previous research [87,88].

The higher risk of mental health outcomes in sexual minorities is due to a variety of reasons. Minority stress seems to have been associated with stressor factors that significantly affect the mental health of MSM. These include internalized homophobia, perceived stigma, and life experiences of discrimination and prejudice [44]. Therefore, it would appear that any of these pressures could greatly affect the mental health and well-being of MSM who practice chemsex. The findings found in the review pertaining to mental health could also be explained by other factors, such as lower educational level, lack of employment, sentimental situation, and younger (and older) age. Note that these factors can also be found in the general population. Stressful experiences in childhood, suffering sexual abuse in childhood, or an unstable family are associated with a multitude of mental health symptoms in MSM to a greater extent than in heterosexual people. These symptoms include psychiatric symptoms, physical illnesses, alcohol and drug use, or experiences of victimization [89].

On the other hand, only some of the articles reviewed established relationships between the use of psychoactive substances for sexual purposes and mental health outcomes. Not all MSM who engage in chemsex experience mental health symptoms; however, a relationship between drug use and mental health may exist in some cases [90,91,92]. The findings of this review suggest that problematic drug use in sexual contexts might only occur in a minority of MSM who practice chemsex. Further, according to Hammoud et al. [63] and Vaccher et al. [58], this could be because some men who ingest drugs for sexual purposes tend to have a greater social commitment with other MSM. This can counteract some of the symptoms related to a worse mental health outcomes. Another possible explanation is provided by Bourne et al. [19] and Weatherburn et al. [93], who reported that some people can perceive positive benefits from drug use, regardless of whether they suffer adverse effects. This is the case in several of the analyzed studies, where some participants, despite having difficulties in achieving and maintaining an erection associated by the consumption of chemsex drugs, consumed other substances (erectile dysfunction drugs) with the objective of counteracting these effects [4,17].

The present review is limited by the small number of studies that have assessed the mental health associated with the use of substances for sexual purposes. Regarding the sample, some studies have been restricted to seropositive men, and others have only studied the use of drugs when they are administered intravenously, which generates certain heterogeneity between the different investigations. On the other hand, the instruments used to assess mental health among MSM who use drugs for sexual purposes are not homogeneous between the different studies, as up to 31 different evaluation measures were analyzed. This can create difficulties when evaluating the results, since it is known that self-report measures have less sensitivity than in-depth clinical evaluations [94]. Furthermore, most of the instruments used are batteries or short screening tests. While this is because they are easy to apply and generally require a limited amount of time, it could lead to a skewed estimation in the association of the variables studied. To improve the study and understanding of mental health symptoms associated with chemsex, it would be interesting to have a standardized instrument that assesses the behavioral and psychological aspects of chemsex phenomenon in detail.

Some MSM who participate in chemsex make use of several substances. Therefore, the mental health symptoms could be associated with polydrug use rather than with ingestion of a single drug for sexualized use. Accordingly, it would be interesting to continue studying consumption patterns among those who practice chemsex. Other limitations of this study are the lack of longitudinal investigations that include the temporal sequence of chemsex practice and the lack of an adequate control group in several analyzed studies.

Finally, it would be interesting to evaluate the incidence of chemsex in smaller cities to understand its prevalence and idiosyncrasies. Currently, there is empirical evidence to suggest that cognitive functioning is an important factor in the acquisition and maintenance of addictive behavior [95]. Therefore, we also recommend future research to explore different neurocognitive outcomes (such as inhibitory control or working memory) and their association with sexualized drug use. Finally, future lines of research and the practical implications of the results should be developed more fully.

## 5. Conclusions

The relationship between mental health and chemsex continues to be poorly addressed and there is a great heterogeneity in terms of the type of substances analyzed. The main mental health symptoms of people who practice chemsex are depression and anxiety symptoms, although other outcomes have also been described with less evidence. People who choose slamsex as a route of administration exhibit more acute mental health outcomes. We cannot draw far-reaching conclusions linking sexualized drug use to poorer mental health, and more comprehensive evaluations of the psychological profiles of those who wish to practice chemsex should be established. In addition, the implementation of psychoeducation and psychological care strategies based on individual parameters is important as a measure to avoid the exacerbation of mental health symptoms among MSM who practice chemsex.

## Figures and Tables

**Table 1:** Search strategy used.

Population	Intervention	Outcome
Men who have sex with men	Sexualized drug	Psychological disorders
(MSM) Gay man/men Bisexual man/men Homosexual man/men	Sexualized drug use Sexualized substance Slamsex Party and play Chemsex Mephedrone Cathinones N-methyl-3,4-methylenedioxyamphetamine MDMA GHB Gamma hydroxybutyrate Gamma hydroxybutyric acid Ketamine Cocaine Poppers Methamphetamine Viagra	Psychiatric disease Mental disorders Mental health Mental health symptoms Mental health outcomes Psychological wellbeing Anxiety Depression Addictive Behavior

**Table 2:** Descriptive characteristics of the included studies.

Author and Year	Study	Title	Year of Study	Journal	Country	Objective	Study Design	n (Age)
(Batisse et al., 2016) [59]	-	Use of psychostimulants in a sexual context: Analysis of cases reported to the French network of Addictovigilance Centers	2016	Therapies	France	Estimate prevalence of psychiatric disorders, intoxication, dependence and substance abuse in people who practice slamsex.	Cross-sectional	51 (Mean = 40)
(Brogan et al., 2019) [22]	Canadian EMIS-2017	Canadian results from the European Men-who-have-sex-with-men Internet survey (EMIS-2017)	2019	Canada Communicable Disease Report	Canada	Assess needs related to sexually transmitted infections of gays, bisexuals, and other men who have sex with men.	Cross-sectional	5165 (Median = 36)
(Card et al., 2019) [60]	Momentum Health Study	Escape expectancies and sexualized substance use among gay, bisexual, and other men who have sex with men	2019	AIDS care	Canada	Examine how McKirnan’s Cognitive Escape Theory (CES) is related to the use of sexualized substances.	Cross-sectional	774 (Range = 25–47)
(Demant & Oviedo-Trespalacios, 2019) [61]	-	Harmless? A hierarchical analysis of poppers use correlates among young gay and bisexual men	2019	Drug and Alcohol Review	Australia	Examine recent poppers use patterns with personal characteristics, other substance use, as well as mental and psychosocial health.	Cross-sectional	836 (Mean = 23.4)
(Dolengevich-Segal et al., 2019) [62]	U-SEX GESIDA 9416 study	Drug-related and psychopathological symptoms in HIV-positive men who have sex with men who inject drugs during sex (slamsex): Data from the U-SEX GESIDA 9416 Study	2019	Plos One	Spain	Describe the physical and psychopathological symptoms of sexualized intravenous drug use (slamsex).	Cross-sectional	742 (Range = 33–44)
(Hammoud et al., 2017) [63]	Following Lives Undergoing Change (Flux) Study	Intensive sex partying with gamma-hydroxybutyrate: factors associated with using gamma-hydroxybutyrate for chemsex among Australian gay and bisexual men—results from the Flux Study	2017	Sexual Health	Australia	To study factors associated with the use of GHB and its relationship with sex: risk behavior, contexts, consequences and motivations for its use.	Prospective observational	3190 (Mean = 35)
(Hibbert et al., 2019) [64]	-	Psychosocial and sexual characteristics associated with sexualized drug use and chemsex among men who have sex with men (MSM) in the UK	2019	Sexually Transmitted Infections	United Kingdom	To study psychosocial and sexual patterns of the use of sexualized drugs in men who have a relationship with other men.	Cross-sectional	3676 (Mean = 30.7)
(Nöstlinger et al., 2020) [65]	Be-PrEP-ared	Drug use, depression and sexual risk behavior: a syndemic among early pre-exposure prophylaxis (PrEP) adopters in Belgium?	2020	AIDS Care	Belgium	To assess the interaction of drug use and depression with risky sexual behavior.	Longitudinal, prospective cohort study	Baseline (200), M9 (186) and M18 (179) (Range = 22–70)
(Schecke et al., 2019) [66]	German Chemsex Survey	Crystal Methamphetamine use in sexual settings among German men who have sex with men	2019	Frontiers in Psychiatry	Germany	To study the use of methamphetamine in sexual settings and its association with the acquisition and transmission of sexually transmitted infections.	Cross-sectional	1050 (Mean = 34.5)
(Sewell et al., 2017) [14]	AURAH	Poly drug use, chemsex drug use, and associations with sexual risk behavior in HIV-negative men who have sex with men attending sexual health clinics	2017	International Journal of Drug Policy	England	To assess prevalence of multiple drug use and chemsex practice and its association with risky sexual behavior.	Cross-sectional	1484 (Median = 31.5)
(Trouiller et al., 2020) [67]	PREVAGAY 2015 bio-behavioural survey	Injecting drug use during sex (known as “slamming”) among men who have sex with men: Results from a time-location sampling survey conducted in five cities, France	2020	International Journal of Drug Policy	France	Estimate prevalence of men who practice slamsex and identify factors associated with this practice.	Cross-sectional	2610 (Range = 28.7 –43)
(Vaccher et al., 2020) [58]		Prevalence, frequency, and motivations for alkyl nitrite use among gay, bisexual and other men who have sex with men in Australia	2020	International Journal of Drug Policy	Australia	To determine the prevalence and frequency of popper use and the factors associated with its use and to examine the motivations for using poppers.	Prospective observational	3273 (Range = 25–46)

-: no data.

**Table 3:** Consumer drugs in included studies.

Author and Year	Chemsex	Drugs	Cannabis	Poppers	GHB/ GBL	Mephedrone	Speed/ Meth	Ketamine	MDMA	Viagra	Cocaine	Slamming
Batisse et al. (2016) [59]	90%	Of those who practice slamming	11%	11%	13%	**	8%	8%	6%	-	33%	* 60.78%
Brogan et al. (2019) [22]	5.8% (previous 6 months)	Of the entire sample	46.6%	-	7.35%	0.40%	6.1 %	-	8.7%	-	14%	3.5%
Card et al. (2019) [60]	11.59% (previous 6 months)	Of the entire sample	-	-	-	-	-	-	-	-	-	-
Demant & Oviedo-Trespalacios, (2019) [61]	-	Of the entire sample	-	* 43.8%	-	-	-	-	-	-	-	-
Dolengevich-Segal et al. (2019) [62]	29.11% (previous 12 months)	From chemsex group	-	78.7%	71.7%	69.4%	29.6%	36.1%	48.6%	-	45.4%	* 15.7%
Hammoud et al. (2017) [63]	16.9% (previous 6 months)	Of the entire sample	30.0%	35.1%	* 5.4%	-	12%	-	17.7%	36.4%	-	-
Hibbert et al. (2019) [64]	6% (previous 12 months)	From sexualized drug use sample	13%	28%	3%	3%	1%	2%	4%	12%	10%	-
Nöstlinger et al. (2020) [65]	Baseline: 45% (previous 3 months)	*Of the entire Sample (baseline)*	-	-	38.5%	7.5%	15%	34.5%	42.5%	-	30%	-
Schecke et al. (2019) [66]	12.4% (previous 12 months)	Of those who used methamphetamine	51.5%	93.8%	70.8%	40.8%	*	53.8%	62.3%	76.2%	46.9%	-
Sewell et al. (2017) [14]	21.8% (previous 3 months)	Of the entire sample	21.0%	32.9%	12.0%	19.1%	6.4%	8.4%	13.0%	17.1%	19.4%	2%
Trouiller et al. (2020) [67]	20.8% (previous 12 months)	Of those who practice slamming	-	-	49%	55%	43%	-	-	-	43%	* 3.1%
Vaccher et al. (2020) [58]	26.3% (previous 6 months)	Of those who used poppers	-	* 45.9%	16.5%	-	20%	-	33.1%	-	28.4%	-

*: drug or testing practice in the study; **: refers to being the most consumed but does not indicate how much; -: no data. GHB/GBL: Gamma hydroxybutyrate/Gamma hydroxybutyric; Meth: Methamphetamine.

**Table 4:** Psychological instruments included.

	(Batisse et al., 2016) [59]	(Brogan et al., 2019) [22]	(Card et al., 2019) [60]	(Demant & Oviedo-Trespalacios, 2019) [61]	(Dolengevich-Segal et al., 2019) [62]	(Hammoud et al., 2017) [63]	(Hibbert et al., 2019) [64]	(Nöstlinger et al., 2020) [65]	(Schecke et al., 2019) [66]	(Sewell et al., 2017) [14]	(Trouiller et al., 2020) [67]	(Vaccher et al., 2020) [58]
PHQ-4		*										
PHQ-9								*				
PHQ-15									*			
CAGE-4		*										
EMS			*									
HADS			*									
SSSS			*									
SAS			*									
SOS			*									
ASSIST				*								
PSOC-LGBT				*								
DTCQ				*								
SUMS				*								
MHC				*								
K10				*			*					
BRS				*								
MSS				*								
CISS				*								
AUDIT-C				*						*		
SMSE						*						
GAD-7						*			*	*		*
IHS							*					
OBCS							*					
UCLA							*					
SWLS							*					
RSE												
SDS												
PC-PTSD									*			
SF-36											*	
GSE												*
QQ					*							
SS					*							
SPB	*											

* Used instrument; PHQ = Patient Health Questionnaire; CAGE-4 = CAGE questionnaire; EMS = Escape Motivation Scale; HADS = Hospital Anxiety and Depression scale; SSSS = Sexual Sensation Seeking Scale; SAS = Sexual Altruism Scale, SOS = Scale of Optimism-Skepticism; ASSIST = Alcohol, Smoking and Substance Involvement Screening Test; PSOC-LGBT = Psychological Sense of LGBT Community Scale; DTCQ = Drug-Taking Confidence Questionnaire; SUMS = Substance Use Motives Scale; MHC = Mental Health Continuum; K10 = Kessler-10 Psychological Distress Scale; BRS = Brief Resilience Scale; MSS = Minority Stress Scales; CISS = Coping Inventory of Stressful Situations; AUDIT-C = Alcohol Use Disorder Identification Test C; SMSE = Scale Measuring Social Engagement; GAD-7 = Generalized Anxiety Disorder Assessment; his = Internalized Homophobia Scale; OBCS = Objectified Body Consciousness Scale; UCLA = UCLA Loneliness Scale; SWLS = Satisfaction With Life Scale; PC-PTSD = Primary Care PTSD Screen; SF-36 = Short Form Health Survey; GSE = Gay social engagement; QQ = Questions in the Questionnaire on previously diagnosed psychiatric disorders; SS = self-reported symptoms; SPB = Self-reported Psychological Background.

## Data Availability

Not applicable.
